# Supplementary material for: Understanding Editing Behaviors in Multilingual Wikipedia
Source: PLoS One. 2016 May 12;11(5):e0155305. doi: 10.1371/journal.pone.0155305 (PMC4865083; doi:10.1371/journal.pone.0155305)
Supplement: S1 Table — Examples of English Wikipedia article titles for discovered topics. (PDF) [file pone.0155305.s001.pdf]

Supporting Information

S1 Table

Topic 1: Science	Topic 2: Football
Museum Boerhaave	Bebé
History of photography	Georgi Kinkladze
Planetarium	Raïs M'Bolhi
List of Dutch inventions and discoveries	John Carew
Electrotyping	John Guidetti
**Topic 3: Film**	**Topic 4: Middle East Geography**
Levar Burton	Counties of Iran
Tribeca Film Festival	Shevir
Romeo and Juliet (films)	Robat (disambiguation)
Viggo Mortensen	Rijan
Walt Disney	Chalmeh
**Topic 5: American Sports**	**Topic 6: Songs & Albums**
October 2005 in sports	Hurry Up, We're Dreaming
NHL trade deadline	Paradise (Lana Del Rey EP)
2005 Nebraska Cornhuskers baseball team	The Sweet Escape
Bobby Ryan	Ceremonials
History of the New York Jets	Do It Again (the Beach Boys Song)
**Topic 7: Musicians**	**Topic 8: Cities**
The Decemberists	Schüttorf
The Band	Rodgau
Carlos Santana	Aachen
Brian May	Olpe
Dirty Three	Lisbon
**Topic 9: Global Sports**	**Topic 10: TV Shows**
List of association football competitions	The Hunger Games Trilogy
1982 FIFA World Cup	The Penguins of Madagascar
Promotion and relegation	The Real Adventures of Jonny Quest
Lokomotiv Cove FC	The Animatrix
1998 FIFA World Cup	M.I. High
**Topic 11: Politics**	**Topic 12: History**

European Conservatives and Reformists	Pierre-Marie-Alphonse Favier
Viktor Yushchenko	Society of the Song Dynasty
List of state leaders in 1993	Taukei Ni Waluvu
Tarja Halonen	17th century
United Kingdom Alternative Vote Referendum	Eruera Maihi Patuone
**Topic 13: Military**	**Topic 14: Transportation**
Air Warfare of World War II	Railway station layout
Battle of Jutland	Railways in Melbourne
Defence of the Reich	LSWR suburban lines
Battle of the River Plate	Train station
A World at War	Hastings Line
**Topic 15: Computer**	**Topic 16: Education**
Packet switching	Stanford University
Videoconferencing	Waded Cruzado
List of software forks	Massachusetts Institute of Technology
Bulletin board system	Vishen Lakhiani
Trusted computing	University of California, Berkeley
**Topic 17: Geographical Locations**	**Topic 18: Descriptive**
Quehanna Wild Area	BBC Sport
Global storm activity of 2009	BMC Racing Team
Death Valley National Park	Coca-Cola formula
Kimberley (Western Australia)	Coal
Hwange National Park	Colombian cuisine
**Topic 19: Olympics**	**Topic 20: Animals & Plants**
Chronological summary of the 2012 Summer Olympics	Lobatus Gigas
2013 IPC Athletics World Championships	List of recently extinct birds
2014 in Sports	Galápagos Tortoise
Yelena Isinbayeva	Common Starling
1980 Summer Olympics	Giant Trevally

Topic Clusters from the English Edition of Wikipedia. Examples of English Wikipedia article titles for discovered topics.
