# Supplementary material for: Understanding Editing Behaviors in Multilingual Wikipedia
Source: PLoS One. 2016 May 12;11(5):e0155305. doi: 10.1371/journal.pone.0155305 (PMC4865083; doi:10.1371/journal.pone.0155305)
Supplement: S2 Table — Examples of Spanish Wikipedia article titles for discovered topics (presented in English). (PDF) [file pone.0155305.s002.pdf]

Supporting Information

S2 Table

Topic 1: Art	Topic 2: Descriptive
Kinetic art	Ancient history
Self-portrait	Psychokinesis
Claude Monet	European Train Control System
History of architecture	Functional programming
São Paulo Museum of Art	Pluto
**Topic 3: Soccer**	**Topic 4: Film**
Celtic F.C.	The Great Gatsby (1974 film)
Carlos Tévez	Tom Hiddleston
1964 Argentine Primera División	Nickelodeon Movies
Everton F.C.	Penélope Cruz
FC Barcelona	Johnny Depp
Kaley Cuoco	Kaley Cuoco
**Topic 5: Animal**	**Topic 6: Global Sports**
Nudochernes leleupi	Asian Football Confederation
Oxydactylus	2013 FIFA Confederations Cup
Mordellistena chopardi	2018 FIFA World Cup
Parazaona pycta	UEFA Women's Euro 2013
Desert Sparrow	Argentina national rugby union team
**Topic 7: History**	**Topic 8: Plants**
Gustaf VI Adolf of Sweden	Notholaena
Hundred Years' War	Martretia
King in Prussia	Peach
Catholic Monarchs	Trevo
Maximilian I of Mexico	Totora (plant)
**Topic 9: Politicians**	**Topic 10: Natural Science**
Alfonso Guerra	Ozone layer
List of Governors of Iowa	Nitrogen cycle
First Lady of Peru	Biomechanics
Ban Ki-moon	Earth's rotation
President of Honduras	Spinal cord

**Topic 11: Social Science**	**Topic 12: Music**
Democratic centralism	Red (Taylor Swift album)
Political economy	Boys Like Girls
Market research	Barcelona (song)
Social movement	Get Lucky (Daft Punk song)
The Third Wave	Folk music
**Topic 13: Cities**	**Topic 14: Geographical Locations**
Machu Picchu	Regions of Senegal
Madrid	Guavio Province
New Delhi	Central Region Venezuela
Montreal	Province of Castellón
Santiago	Khar Turan National Park
**Topic 15: Olympics**	**Topic 16: Literature**
Czechoslovakia at the 1988 Winter Olympics	Spanish Golden Age
Denmark at the 1992 Winter Olympics	Voltaire
Great Britain at the 1992 Winter Olympics	Poetry
Finland at the 2010 Winter Olympics	Franz Kafka
Hungary at the 1956 Winter Olympics	Giovanni Papini
**Topic 17: Musicians**	**Topic 18: Politics**
Metallica	Venezuelan Democratic Party
Billy Joel	Social Christian Reformist Party
Janis Joplin	Andalusian parliamentary election 2004
Keith Emerson	United States presidential election 1788–1789
Marilyn Manson	Green Ecologist Party (Chile)
**Topic 19: Entertainment**	**Topic 20: Tennis**
Half-Life 2	2013 ATP Challenger Tour
Glee (TV series)	Canadian Open (tennis)
The Phantom of the Opera (1986 musical)	2013 Western & Southern Open
Garfield	Maria Sharapova
Nickelodeon	Rafael Nadal

Topic Clusters from the Spanish Edition of Wikipedia. Examples of Spanish Wikipedia article titles for discovered topics (presented in English).
