# Supplementary material for: Understanding Editing Behaviors in Multilingual Wikipedia
Source: PLoS One. 2016 May 12;11(5):e0155305. doi: 10.1371/journal.pone.0155305 (PMC4865083; doi:10.1371/journal.pone.0155305)
Supplement: S3 Table — Examples of German Wikipedia article titles for discovered topics (presented in English). (PDF) [file pone.0155305.s003.pdf]

Supporting Information

S3 Table

**Topic 1: Computer**	**Topic 2: Natural Science**
IPv6 rapid deployment	Boiling point
C (programming language)	Solar cell
Real-time operating system	Recombinant DNA
Graphical user interface	Klinefelter syndrome
Web Server Gateway Interface	Volcano
**Topic 3: Descriptive**	**Topic 4: Geographical Locations: U.S.**
Abraham Lincoln	Moorhead Mississippi
Anti-Zionism	Madison County Texas
Ice	List of cities in Virginia
Eiffel Tower	Leon County Florida
BMW 340	San Rafael California
**Topic 5: Names**	**Topic 6: Geographical Locations: Europe**
Watson (Surname)	Westfalen
Williamson (Surname)	Pram Austria
Taylor (Name)	Monforte Portugal
Shaw (Name)	Hamburg-Marienthal
Peter (Given Name)	Cologne (Region)
**Topic 7: History**	**Topic 8: Academic**
List of state leaders in 655	University of Mannheim
Charles VIII of Sweden	Computer science
Frederick William I of Prussia	Individual psychology
Vianden Castle	Industrial sociology
Guelderian Wars	Keynesian economics
**Topic 9: Celebrities**	**Topic 10: Soccer**
List of American novelists	List of football clubs in Germany
Lists of golfers	2003–04 UEFA Cup
List of Medal of Honor recipients	Real Madrid C.F.
Franklin Roosevelt	Lionel Messi
Sebastian Shaw	Son Heung-Min

**Topic 11: Cultural Heritage**	**Topic 12: Musicians**
List of Cultural Property in Oberwil-Lieli	Eminem
List of properties in Krumbach	Flo Rida
List of properties in Gleisdorf	2012 Grammy Awards
List of properties in Arnoldstein	John Lennon
List of properties in Bürs	Lady Gaga
**Topic 13: Natural Topography**	**Topic 14: Land Transport**
Najerilla (River)	Hesper Valley Railway
Olympic National Park	Innsbruck Central Station
Nock Mountains	Highways in Bulgaria
Niedersonthofener See	List of tunnels in Germany
Quincy Bay	Metrobus
**Topic 15: Politicians**	**Topic 16: Entertainment**
Rudolf Hierl (Politician)	Denzel Washington
Ulrich Kelber	Bradley Cooper
Robert Thaller	Anna Karenina (2012 Film)
Norbert Otto (Politician)	Braid (Video Game)
Liam Aylward	Boss (TV series)
**Topic 17: Air transport**	**Topic 18: Global Sports**
Lombok International Airport	Badminton at the Asian Games
Salzburg Airport	Australian Football International Cup
Airline	Australian Goldfields Open
Aviation accidents and incidents	Australia national association football team
Human-powered helicopter	2012–13 FA Cup
**Topic 19: Authors**	**Topic 20: Military**
Franz Kafka	Japanese conquest of Burma
Jan Zimmermann	Pacific War
Herbert Schmidt	Russian Navy
List of authors by name: F	Soviet submarine K-19
List of authors by name: W	Rocket artillery

Topic Clusters from the German Edition of Wikipedia. Examples of German Wikipedia article titles for discovered topics (presented in English).
