# Supplementary material for: Understanding Editing Behaviors in Multilingual Wikipedia
Source: PLoS One. 2016 May 12;11(5):e0155305. doi: 10.1371/journal.pone.0155305 (PMC4865083; doi:10.1371/journal.pone.0155305)
Supplement: S4 Table — Syntactic complexity for 20 English, Spanish and German topics and independent t-test results comparing contributions by primary and non-primary editors. (PDF) [file pone.0155305.s004.pdf]

Supporting Information

S4 Table

Topic	Complexity	Primary	Non-Primary	t	p-value
Science	2.917	0.064	0.061	0.747	4.550E-01
Football	1.995	0.029	0.031	-0.756	4.496E-01
Film	2.610	0.046	0.048	-0.542	5.880E-01
Middle East Geography	1.733	0.001	0.002	-1.081	2.799E-01
American Sports	2.312	0.006	0.007	-0.361	7.184E-01
Music: Song, Albums	2.495	0.030	0.026	1.343	1.793E-01
Music: Musician	2.630	0.057	0.060	-0.801	4.231E-01
Cities	2.638	0.043	0.057	-4.023	5.796E-05
Sports Related Articles	1.563	0.047	0.048	-0.122	9.029E-01
TV Shows	2.930	0.056	0.059	-0.840	4.009E-01
Politics	2.701	0.030	0.027	0.955	3.396E-01
History	3.071	0.196	0.189	1.208	2.271E-01
Military	2.938	0.033	0.034	-0.536	5.919E-01
Transportation	2.565	0.014	0.016	-1.368	1.715E-01
Computer	2.577	0.057	0.049	2.099	3.582E-02
Education	2.776	0.078	0.074	0.846	3.975E-01
Geographical Locations	2.676	0.023	0.019	1.753	7.960E-02
Descriptive	2.505	0.152	0.151	0.129	8.974E-01
Olympics	1.952	0.022	0.023	-0.348	7.278E-01
Animal, Plants	2.770	0.017	0.020	-1.471	1.413E-01

Table 1: **Syntactic Complexity for each Topic by Language Edition.** Syntactic complexity for 20 English topics and independent t-test results comparing contributions by primary and non-primary editors.

Topic	Complexity	Primary	Non-Primary	t	p-value
Computer	3.219	0.047	0.030	3.070	2.157E-03
Natural Science	3.511	0.035	0.022	2.943	3.276E-03
Descriptive	3.220	0.304	0.322	-1.445	1.486E-01
Geographical Loc.: U.S.	2.025	0.002	0.002	-0.434	6.643E-01
Names	2.361	0.013	0.011	0.700	4.840E-01
Geographical Loc.: Europe	2.964	0.036	0.028	1.685	9.200E-02
History	3.341	0.046	0.037	1.619	1.055E-01
Academic	3.432	0.123	0.117	0.709	4.783E-01
Celebrities	3.120	0.038	0.036	0.392	6.949E-01
Soccer	2.769	0.036	0.051	-2.393	1.679E-02
Cultural Heritage	1.906	0.004	0.001	2.404	1.629E-02
Musicians	3.034	0.049	0.053	-0.483	6.292E-01
Natural Topography	3.062	0.025	0.025	0.019	9.845E-01
Land Transport	3.306	0.026	0.027	-0.139	8.896E-01
Politicians	3.207	0.056	0.067	-1.756	7.921E-02
Entertainment	3.331	0.052	0.061	-1.408	1.591E-01
Air Transport	3.291	0.017	0.016	0.194	8.461E-01
Global Sports	2.531	0.024	0.035	-2.173	2.987E-02
Authors	3.429	0.046	0.037	1.765	7.760E-02
Military	3.630	0.021	0.021	-0.050	9.603E-01

Table 2: **Syntactic Complexity for each Topic by Language Edition.** Syntactic complexity for 20 German topics and independent t-test results comparing contributions by primary and non-primary editors.

Topic	Complexity	Primary	Non-Primary	t	p-value
Art	3.012	0.016	0.016	0.025	9.804E-01
Descriptive	2.808	0.384	0.418	-2.159	3.092E-02
Soccer	2.347	0.077	0.072	0.593	5.536E-01
Film	2.747	0.038	0.038	0.064	9.494E-01
Animal	2.444	0.002	0.002	-0.358	7.201E-01
Global Sports	2.295	0.038	0.031	1.115	2.648E-01
History	3.001	0.011	0.010	0.417	6.769E-01
Plants	2.376	0.005	0.017	-3.429	6.189E-04
Politicians	3.179	0.032	0.019	2.443	1.466E-02
Natural Science	3.084	0.039	0.051	-1.676	9.387E-02
Social Science	3.005	0.042	0.030	2.023	4.322E-02
Music	2.789	0.052	0.039	1.741	8.183E-02
Cities	2.931	0.081	0.087	-0.670	5.029E-01
Geographical Locations	2.523	0.020	0.037	-3.324	8.991E-04
Olympics	1.970	0.001	0.002	-0.423	6.725E-01
Literature	3.094	0.033	0.037	-0.645	5.193E-01
Musicians	2.814	0.027	0.024	0.503	6.147E-01
Politics	3.029	0.022	0.019	0.599	5.492E-01
Entertainment	2.967	0.076	0.046	3.736	1.921E-04
Tennis	2.654	0.004	0.005	-0.552	5.811E-01

Table 3: **Syntactic Complexity for each Topic by Language Edition.** Syntactic complexity for 20 Spanish topics and independent t-test results comparing contributions by primary and non-primary editors.
